# Use of QuantiFERON®-TB Gold in-tube culture supernatants for measurement of antibody responses

**DOI:** 10.1371/journal.pone.0188396

**Published:** 2017-11-21

**Authors:** Simon G. Kimuda, Irene Andia-Biraro, Moses Egesa, Bernard S. Bagaya, John G. Raynes, Jonathan Levin, Alison M. Elliott, Stephen Cose

**Affiliations:** 1 Department of Medical Microbiology, School of Biomedical Sciences, Makerere University College of Health Sciences, Kampala, Uganda; 2 MRC/UVRI Uganda Research Unit on AIDS, Entebbe, Uganda; 3 Department of Immunology and Molecular Biology, School of Biomedical Sciences, Makerere University College of Health Sciences, Kampala, Uganda; 4 Department of Internal Medicine, School of Medicine, Makerere University College of Health Sciences, Kampala, Uganda; 5 London School of Hygiene & Tropical Medicine, London, United Kingdom; Fundació Institut d’Investigació en Ciències de la Salut Germans Trias i Pujol, Universitat Autònoma de Barcelona, SPAIN

## Abstract

QuantiFERON®-TB Gold in-tube (QFT-GIT) supernatants may be important samples for use in assessment of anti-tuberculosis (TB) antibodies when only limited volumes of blood can be collected and when a combination of antibody and cytokine measurements are required. These analytes, when used together, may also have the potential to differentiate active pulmonary TB (APTB) from latent TB infection (LTBI). However, few studies have explored the use of QFT-GIT supernatants for investigations of antibody responses. This study determined the correlation and agreement between anti-CFP-10 and anti-ESAT-6 antibody concentrations in QFT-GIT nil supernatant and serum pairs from 68 TB household contacts. We also explored the ability of *Mycobacterium tuberculosis* (*M*.*tb*) specific antibodies, or ratios of antibody to interferon gamma (IFN-γ) in QFT-GIT supernatants, to differentiate 97 APTB cases from 58 individuals with LTBI. Sputum smear microscopy was used to define APTB, whereas the QFT-GIT and tuberculin skin test were used to define LTBI. There were strong and statistically significant correlations between anti-CFP-10 and anti-ESAT-6 antibodies in unstimulated QFT-GIT supernatants and sera (r = 0.89; p<0.0001 for both), and no significant differences in antibody concentration between them. Anti-CFP-10 & anti-ESAT-6 antibodies differentiated APTB from LTBI with sensitivities of 88.7% & 71.1% and specificities of 41.4% & 51.7% respectively. Anti-CFP-10 antibody/*M*.*tb* specific IFN-γ and anti-ESAT-6 antibody/*M*.*tb* specific IFN-γ ratios had sensitivities of 48.5% & 54.6% and specificities of 89.7% and 75.9% respectively. We conclude that QFT-GIT nil supernatants may be used in the place of sera when measuring antibody responses, reducing blood volumes needed for such investigations. Antibodies in QFT-GIT nil supernatants on their own discriminate APTB from LTBI with high sensitivity but have poor specificity, whereas the reverse is true when antibodies are used in combination with *M*.*tb* specific cytokines. Further antibody and antibody/cytokine combinations need to be explored to achieve better diagnostic accuracy.

## 1.0 Introduction

Almost a quarter the world’s population is estimated to be infected with *Mycobacterium tuberculosis* (*M*.*tb*), the bacterium that causes tuberculosis (TB) [1]. The QuantiFERON®-TB Gold in-tube (QFT-GIT) test is an immunological assay that diagnoses *M*.*tb* infection by detecting *M*.*tb* specific interferon-gamma (IFN-γ) in whole blood culture supernatants [2]. It is able to do so with a high sensitivity but cannot differentiate latent TB infection (LTBI) from active TB disease [3]. In the assay, whole blood is stimulated with the freeze dried antigens, early secretory target (ESAT)-6, culture filtrate protein (CFP)-10 and TB7.7, to elicit *M*.*tb* specific responses. The assay also includes positive (mitogen) controls where whole blood is stimulated with phytohaemagglutinin (PHA), a T cell mitogen, and negative (nil) controls where whole blood is left unstimulated [4]. We propose that after completion of QFT-GIT assays, culture supernatants may still be a valuable resource for the reliable measurement of antibody responses.

Several studies have reported the use of QFT-GIT supernatants for extended cytokine profiling [5–8] but we could only find one that has used these samples for antibody analysis [9]. There is increasing evidence supporting a role for antibody mediated immunity against TB [10,11], and so it is becoming important to look at levels of circulating antibodies in TB patients and their contacts. Furthermore, since antibodies are increased in individuals with active pulmonary TB (APTB) in comparison to those with LTBI they may have the potential to differentiate the two *M*.*tb* infection states [12]. This is particularly important because biomarkers that can distinguish APTB from LTBI may have value as predictors of progression to TB disease. QFT-GIT supernatants may provide an important sample in studies measuring antibody responses for this purpose, in instances where serum or plasma is not routinely available or is in limited quantity. This could be the case in TB studies involving young children from whom limited blood can be obtained, or latent TB diagnostic studies that had not originally set out to look at antibody responses in their study populations.

The concentrations of circulating antibodies *in vivo* are normally investigated by testing serum or plasma. However, it remains unknown whether the concentrations of antibodies in QFT-GIT supernatants are also representative of circulating antibody. Since QFT-GIT supernatants are obtained from 16–24 h whole blood cultures it is possible that this period of culture could be sufficient for the production of antibodies into supernatant by antibody secreting cells that may be present in circulation [13,14]. Additionally, stimulation by antigens in test or mitogen control tubes could lead to the differentiation of memory B cells into antibody secreting cells [15,16], leading to a higher concentration of antibodies in QFT-GIT supernatants compared to sera. We investigated whether the concentrations of anti-mycobacterial IgG antibodies from QFT-GIT nil supernatants and sera were different. Additionally, QFT-GIT nil and mitogen supernatant antibody concentrations were compared to determine the impact of a highly potent cell stimulant such as PHA on antibody levels. We also used these data to evaluate the ability of antibodies in QFT-GIT supernatants to detect APTB disease independently, or in combination with IFN-γ cytokine data, in a study group of APTB cases and individuals with LTBI.

## 2.0 Materials and methods

### 2.1 Study design and participants

This study was of a cross-sectional design and was nested in a larger TB household contact research project based in Kampala, Uganda that set out to determine whether co-infections among household contacts (HHCs) of active pulmonary TB (APTB) cases increased their susceptibility to infection with *M*.*tb* [17]. APTB cases and their HHCs were recruited from Kisenyi & Kitebi municipalities of Kampala district between May 2011 and January 2012. APTB was defined based on a positive result following acid-fast bacilli sputum smear microscopy. APTB cases were only selected if they were above the age of 18 years and had just began anti-TB treatment or had received treatment for less than a month. The HHCs were selected if they lived or shared meals with the APTB cases for two weeks prior to their diagnosis with TB, without any bias towards age or gender. The QFT-GIT test was used in conjunction with the tuberculin skin test (TST) to determine *M*.*tb* infection among HHCs with no signs or symptoms of TB, with those who were positive on both tests classified as having a latent TB infection (LTBI), and those who tested negative on both tests classified as uninfected.

### 2.2 Samples

Blood was drawn for serum isolation for diagnosis of co-infections and QFT-GIT testing for *M*.*tb* infection, concurrently. We had access to stored paired serum and QFT-GIT nil supernatants from 68 HHCs left over from these investigations, and used them to determine correlation and agreement between antibody concentrations in these two sample types. We also had access to paired QFT-GIT nil and mitogen supernatants from 63 uninfected individuals, 58 individuals with LTBI and 97 APTB cases. We used these samples to determine whether antibody concentrations between samples correlated and agreed well with each other, and whether they could be employed as biomarkers to differentiate APTB from LTBI. This research was exploratory. All individuals were selected based upon availability of sample and information on TB infection status.

### 2.3 Ethical approvals

This study was approved by the Makerere University Higher Degrees Research & Ethics Committee and the Uganda National Council for Science & Technology. All individuals provided informed written consent and they also consented to the use of their samples for future immunological analyses. All samples were fully anonymised before laboratory investigations to protect the study participants’ identities.

### 2.4 Antigens

We used CFP-10 and ESAT-6 of *M*.*tb* (BEI Resources, National Institute of Allergy and Infectious Diseases, National Institutes of Health) for antibody assays. These proteins were chosen because they are encoded by genes in the region of difference (RD)-1 which is present in *M*.*tb* but absent in *Mycobacterium bovis* Bacille-Calmette-Guérin (BCG) as well as most environmental mycobacteria [18]. Immune responses to these proteins are therefore valuable as biomarkers of *M*.*tb* infection.

### 2.5 Antibody ELISA assay

In the procedure, 96 well Immulon® 4 HBX microtitre plates (Thermo Scientific, USA) were coated using 50 μl/well of 2.5 μg/ml CFP-10 or ESAT-6 diluted in bicarbonate coating buffer overnight at 4°C. We also coated the plates with eight, two-fold serial dilutions of human IgG reference standard (Genscript) starting at a concentration of 250 ng/ml. The plates were then washed three times with phosphate saline buffer containing 0.05% tween 20 (PBST) and then blocked with 150 μl/well of 1% skimmed milk for 2 h. A 50 μl volume of serum or supernatant diluted 1:100 in PBST was then added to antigen-coated wells in duplicate while PBST was added to IgG coated wells. Serum-supernatant pairs were run on the same plate and plates were incubated overnight at 4°C. The plates were washed four times in PBST and then 50 μl/well of polyclonal rabbit anti-human IgG conjugated with horseradish peroxidase (Dako, DE) at 0.5 μg/ml in PBST was added to each well and incubated for 1 h at room temperature. The plates were then washed as before and then 50 μl/well of 3,3’,5,5’-tetramethylbenzidine (BD Biosciences-US) was added and the plates developed for 15 min. The optical density (OD) was measured at a wavelength of 450 nm and 630 nm filter as a reference using an ELISA plate reader (Biotek). Human IgG standard concentrations were then used to generate specific antibody concentrations. A pooled sample of QFT-GIT nil supernatants from active TB cases was used as a positive control on each plate. ELISA plate results were only considered to be valid when the positive control antibody concentration was within 3 standard deviations of the mean of the positive control values from all the plate runs.

### 2.6 Data analysis

Data was analysed using Stata release 12.0 statistical package (Statacorp LP, College Station, TX, USA) and GraphPad Prism software, version 6.01 (GraphPad Inc., San Diego, CA, USA). We used Spearman rank correlation to test for associations between the antibody concentrations obtained from assaying paired QFT-GIT supernatants and sera. We used Bland Altman plots [19] to see if there were any differences between log transformed antibody concentrations obtained from sample pairs [20]. For the biomarker analyses, we used the Wilcoxon rank sum test to compare antibody responses or antibody/IFN-γ ratios between groups. We then used receiver operating characteristic (ROC) curves to evaluate ability of these measurements to discriminate APTB cases from the individuals with LTBI. Youden’s index, a measure that provides equal consideration to specificity and sensitivity which is defined as the maximum value of sensitivity + specificity– 1 [21], was used to select optimal antibody or antibody/IFN-γ ratio cut-off levels for positivity from ROC curve analyses.

## 3.0 Results

### 3.1 Demographic characteristics

The median age of the household contacts used for QFT-GIT nil supernatant versus serum comparisons was 14 years (range: 1–66), 44/68 (64.71%) were female, 30/68 (44.1%) were LTBI positive and 6/68 (8.8%) were HIV positive. Characteristics of uninfected controls, individuals with LTBI & APTB cases used for QFT-GIT nil versus QFT-GIT mitogen comparisons and biomarker analyses are described in Table 1. Individuals with APTB & LTBI were older than the uninfected controls but there were no differences in socioeconomic status across the three groups. As expected, there was a higher proportion of males (55.7%; 54/97) and HIV sero-positive individuals (39.2%; 38/97) in the APTB group compared to the LTBI and uninfected control groups [22,23].

**Table 1:** Study participant characteristics.

Characteristic	Uninfected (n = 63)	LTBI (n = 58)	APTB (n = 97)	Total (n = 218)
**Mean age & range (years)**	13 (1, 66)	23 (1, 66)	29 (18, 53)	23 (1, 66)
**Females**	39 (61.9%)	38 (65.5%)	43 (44.3%)	120 (55.1%)
**HIV positive**	3 (4.8%)	5 (8.6%)	38 (39.2%)	46 (21.1%)
**Low SES ^a^**	28 (45.2%)	28 (48.3%)	54 (62.8%)	110 (53.4%)

LTBI = latent tuberculosis infection, SES = socioeconomic status, and APTB = active pulmonary tuberculosis

^a^ Individuals were either of low or medium SES

### 3.2 Agreement between QFT-GIT nil supernatant versus serum and QFT-GIT mitogen versus nil supernatant antibody concentrations

**Fig 1:**
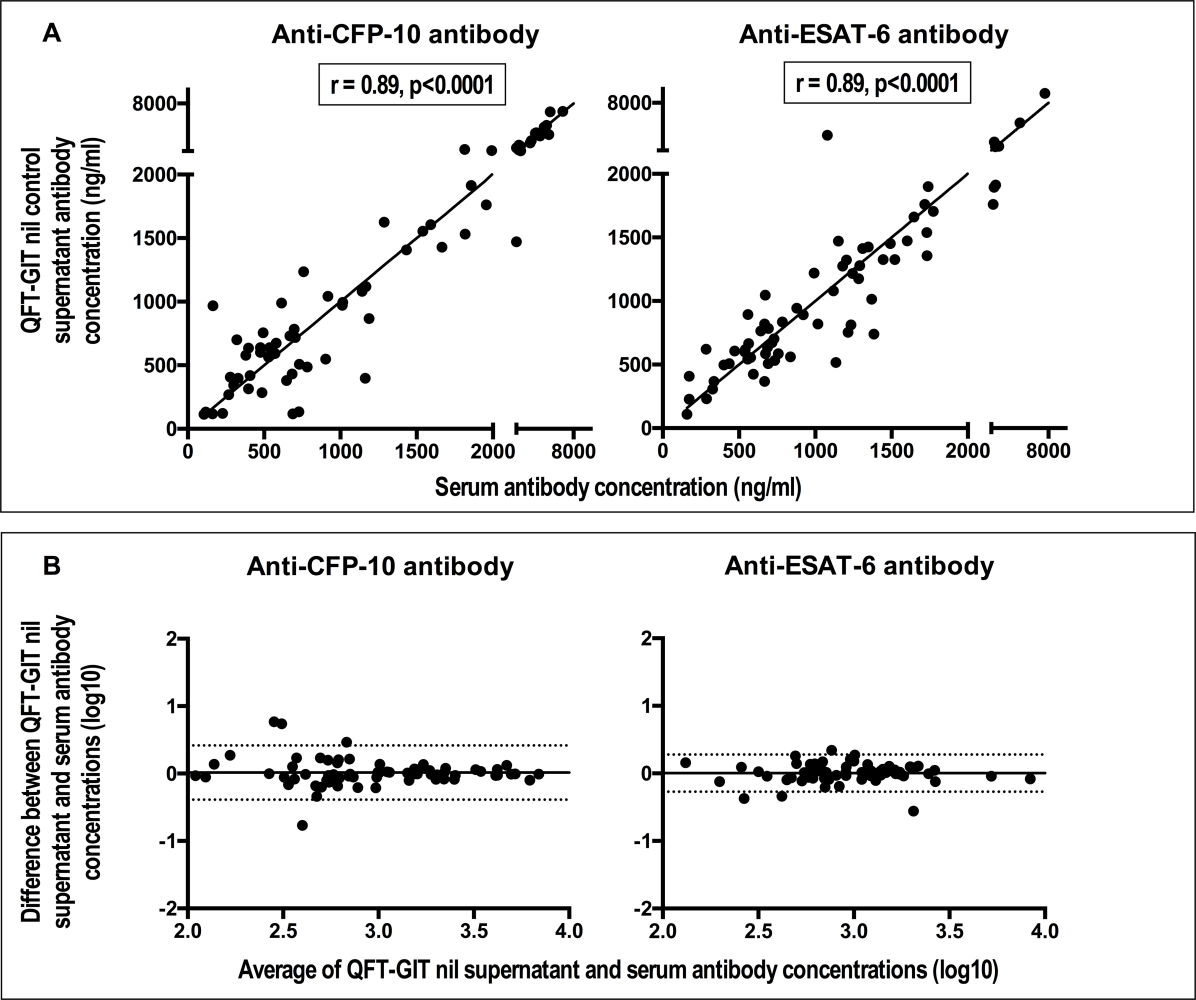
Anti-CFP (culture filtrate protein)-10 & anti-ESAT (early secretory antigenic target)-6 antibody concentrations in QFT-GIT (QuantiFERON®-TB Gold in tube-test) nil control supernatants strongly correlate with those in paired sera and there are no statistical differences between them. Panel A: scatter plots showing correlation. The coefficient (r) and the p values shown correspond to results from Spearman’s rank correlation. The solid lines are lines of identity. Panel B: Bland Altman plots showing agreement. The solid horizontal line represents the bias or average difference while the dotted horizontal lines are 95% confidence intervals.

We compared the concentration of anti-mycobacterial antibodies in paired QFT-GIT nil supernatants and sera from the TB household contacts to determine whether there were differences between the two. There were strong and statistically significant correlations between anti-CFP-10 and anti-ESAT-6 antibody concentrations obtained from assaying QFT-GIT nil supernatants and serum (Fig 1A). In order to assess if this observation was due to agreement between the antibody results from these two samples and was not simply a result of an association, we constructed Bland Altman plots and determined the mean difference in antibody concentrations obtained from assaying QFT-GIT nil supernatants versus serum and its confidence intervals. We found that the mean difference in antibody concentrations obtained from assaying QFT-GIT nil supernatants versus serum was 0.014 (95% CI = -0.388, 0. 416) for anti-CFP-10 antibodies and 0.003 (95% CI = -0.272, 0.278) for anti-ESAT-6 antibodies (Fig 1B). This result implied that there was no evidence of a systematic bias or difference in antibody concentration between the serum and QFT nil supernatant samples and that there is therefore agreement between antibody measurements from the two sample types.

**Fig 2:**
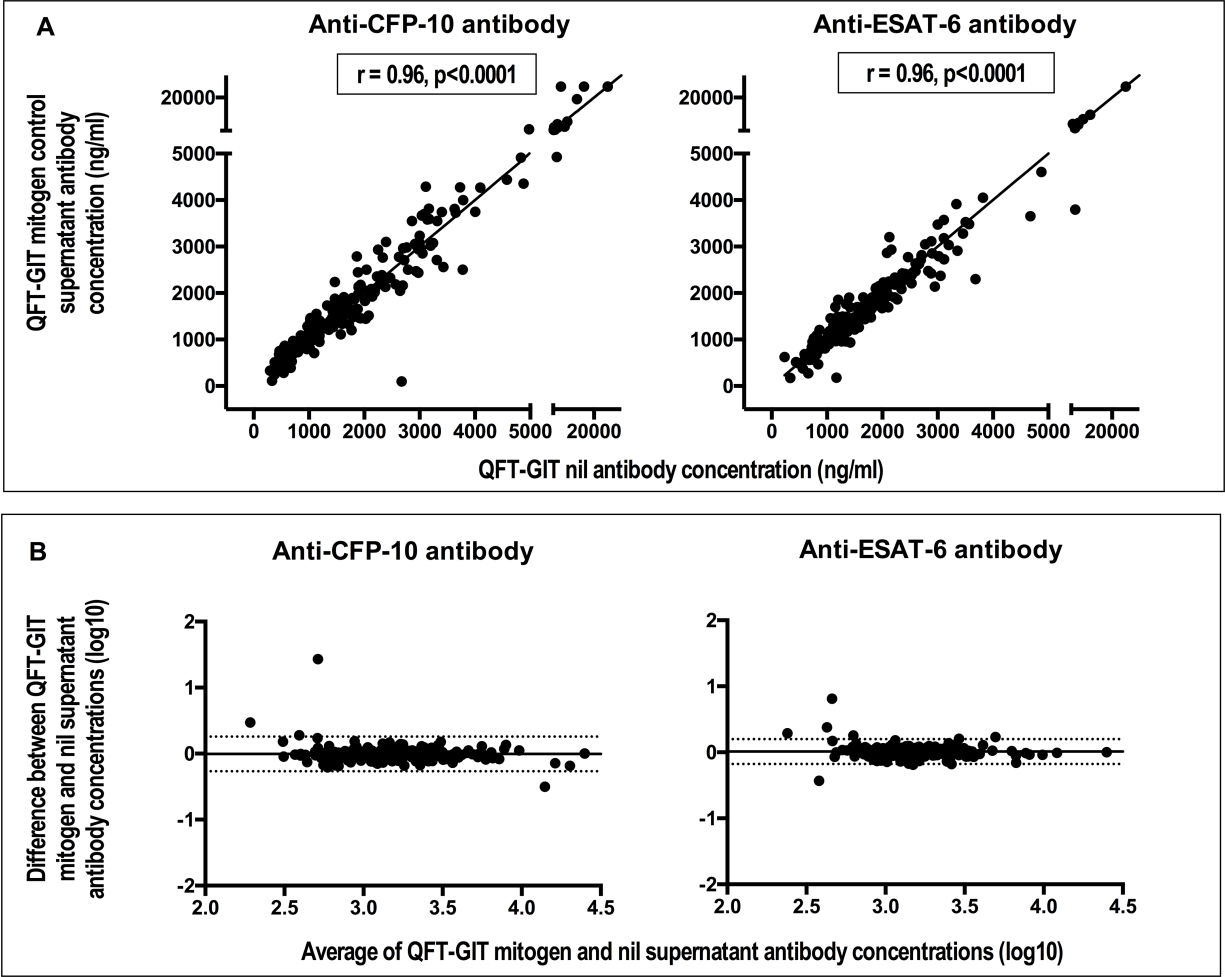
Anti-CFP (culture filtrate protein)-10 & anti-ESAT (early secretory antigenic target)-6 antibody concentrations in QFT-GIT mitogen control supernatants strongly correlate with those in QFT-GIT nil control supernatants and there are no statistical differences between them. Panel A: scatter plots showing correlation. The coefficient (r) and the p values shown correspond to results from Spearman’s rank correlation. The solid lines are lines of identity. Panel B: Bland Altman plots showing agreement. The solid horizontal line represents the bias or average difference while the dotted horizontal lines are 95% confidence intervals.

Supernatants from QFT-GIT stimulated tubes could also have value for antibody analyses. In order to investigate this, paired QFT-GIT mitogen and nil supernatants from uninfected individuals, those with LTBI and APTB cases were tested for antibodies against CFP-10 and ESAT-6 and antibody concentrations from the two sample types compared. There were strong and statistically significant correlations between antibody concentrations from QFT-GIT mitogen and nil supernatants in the individual groups (S1 Fig). Bland Altman analysis showed no evidence of differences between these two QFT-GIT supernatants regardless of the TB infection status of the study participants (S2 Fig). We combined the data from the uninfected individuals, those with LTBI and APTB cases and calculated the overall correlation and agreement. The correlation remained strong and statistically significant (Fig 2A) and Bland Altman analyses continued to show no significant difference: the mean difference in antibody concentrations obtained from assaying QFT-GIT mitogen versus nil supernatants was -0.006 (95% CI = -0.268, 0.257) for anti-CFP-10 antibodies and 0.011 (95% CI = - 0.178, 0.199) for anti-ESAT-6 antibodies (Fig 2B). This meant there was also agreement between QFT-GIT mitogen and nil supernatants antibody concentrations.

### 3.3 Using antibodies in QFT-GIT supernatants to differentiate APTB from LTBI

**Fig 3:**
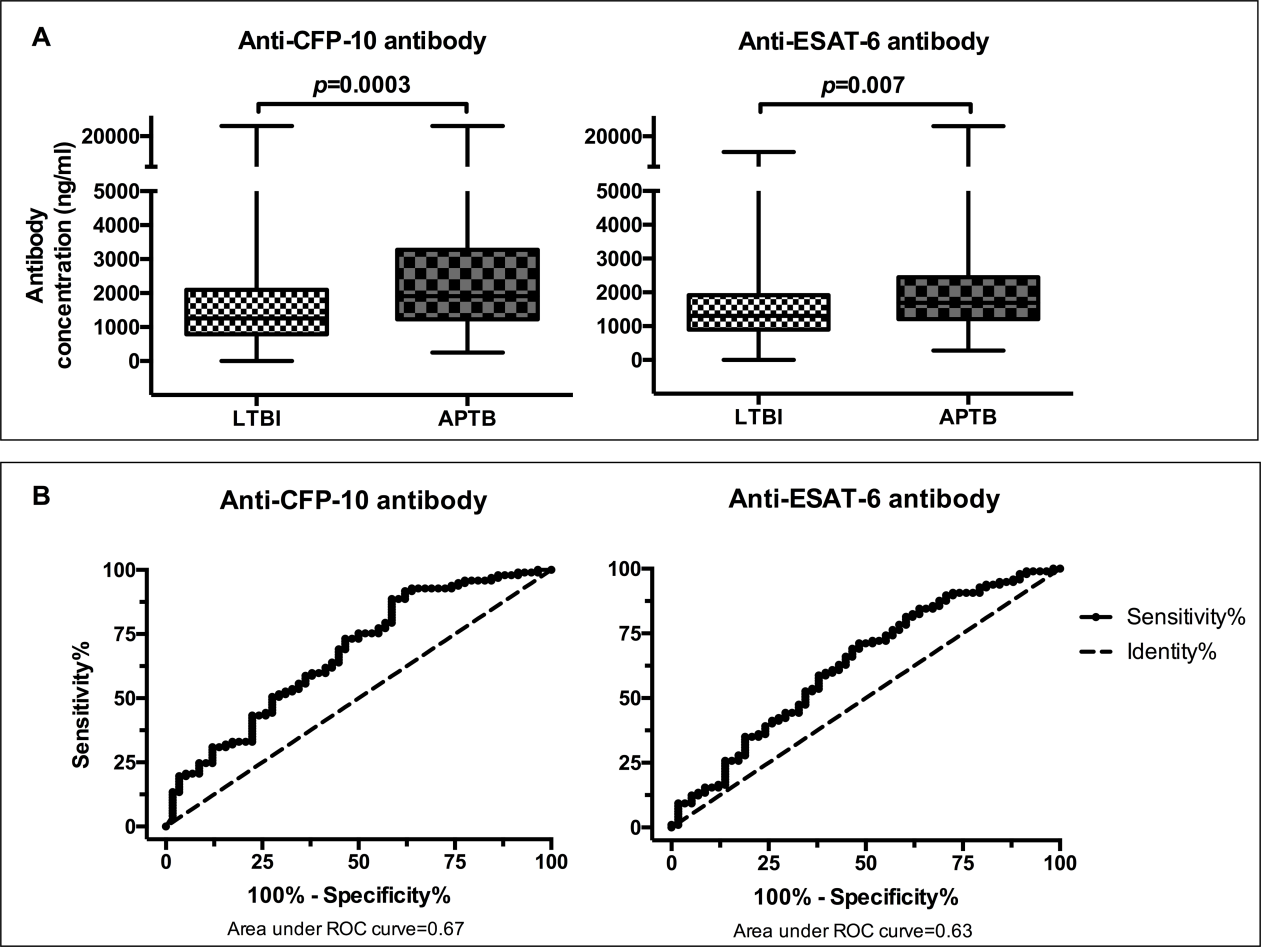
CFP-10 and ESAT-6 specific antibodies in QFT-GIT nil control supernatants from APTB cases & individuals with LTBI and their discriminatory potential. Panel A: box and whisker plots showing differences in antibodies between the two groups. Panel B: Receiver operator characteristic curves showing discriminatory potential at different antibody cut-offs.

Antibodies in QFT-GIT supernatants may have use as biomarkers for the detection of TB disease among *M*.*tb* infected individuals. We therefore determined whether anti-CFP-10 and anti-ESAT-6 antibodies could discriminate APTB cases from individuals with LTBI. In order to investigate this, we used data obtained from testing QFT-GIT nil supernatants from the individuals with LTBI and APTB. APTB cases had higher concentrations of anti-CFP-10 and anti-ESAT-6 antibodies compared to individuals with LTBI (Fig 3A). We found that anti-CFP-10 & anti-ESAT-6 antibodies were able to differentiate APTB from LTBI with an area under the curve of 0.67 (95% CI: 0.58–0.76) & 0.63 (95% CI: 0.54–0.72) respectively (Fig 3B). As mentioned previously, we used Youden’s index to identify the optimal antibody cut-offs for positivity from the ROC curve analyses. This measure gives equal consideration to the values of sensitivity and specificity [21]. Using the cut-offs selected, anti-CFP-10 antibodies differentiated APTB from LTBI with a sensitivity of 87.6% (95% CI: 79.4–93.4) and had a diagnostic specificity of 41.4% (95% CI: 28.6–55.1), whereas anti-ESAT-6 antibodies had a sensitivity of 71.1% (95% CI: 61.0–79.9) and a specificity of 51.7% (95% CI: 38.2–65.0) as shown in Table 2. These findings, taken together, with the area under ROC curve and Youden’s index values indicate that antibodies against CFP-10 differentiated APTB cases from individuals with LTBI with better accuracy compared to antibodies against ESAT-6.

**Table 2:** Potential of anti-CFP-10, anti-ESAT-6 antibodies to differentiate active and latent TB infection.

Biomarker	Cut-off	% Sensitivity (95% CI)	% Specificity (95% CI)	Youden's index (%)
**Anti-CFP-10 antibodies**	998.6 ng/ml	87.6 (79.4–93.4)	41.4 (28.6–55.1)	30
**Anti-ESAT-6 antibodies**	1314.4 ng/ml	71.1 (61.0–79.9)	51.7 (38.2–65.0)	22.9

### 3.4 Using QFT-GIT supernatant antibody/IFN-γ ratio to differentiate APTB from LTBI

**Fig 4:**
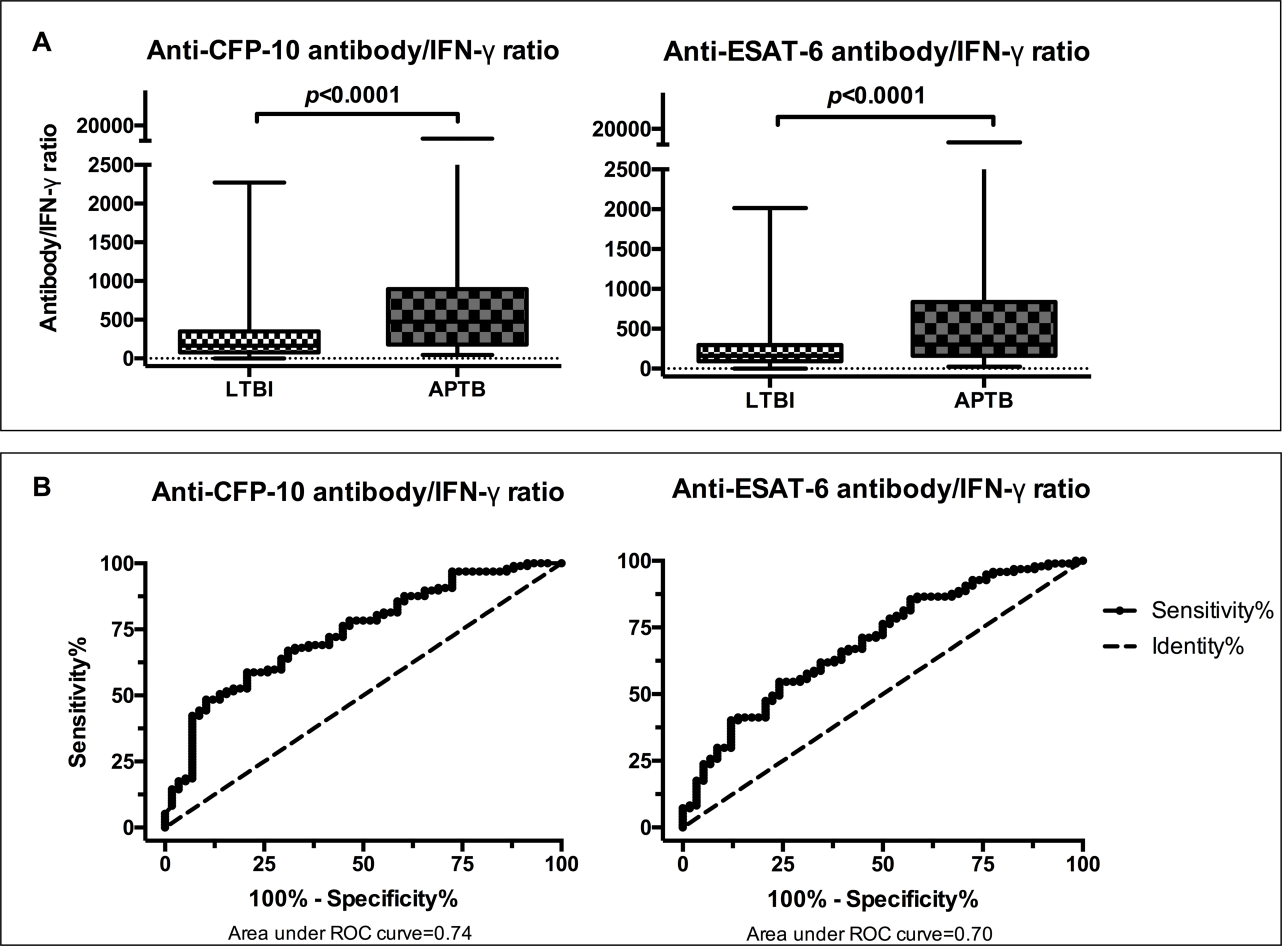
Anti-CFP-10 and anti-ESAT-6 antibody/IFN-γ ratios in APTB cases & individuals with LTBI and their discriminatory potential. Panel A: box and whisker plots showing differences in antibody/IFN-γ ratio between the two groups. The Wilcoxon rank-sum test was used for comparisons. Panel B: Receiver operator characteristic curves showing discriminatory potential at different antibody/IFN-γ ratio cut-offs.

Analysis of QFT-GIT *M*.*tb* specific IFN-γ data generated from the study participants showed that there were higher levels in LTBI compared to APTB (S3 Fig) while our antibody results showed the reverse (Fig 3A). We therefore explored whether antibody/IFN-γ ratio could better discriminate the two groups. We found that these ratios were significantly higher among APTB cases compared to individuals with LTBI (Fig 4A) and so evaluated their diagnostic potential. ROC curve analysis showed that anti-CFP-10 & anti-ESAT-6 antibody/IFN-γ ratios were able to differentiate APTB from LTBI with an area under the curve of 0.74 (95% CI: 0.66–0.82) & 0.70 (95% CI: 0.61–0.78) respectively (Fig 4B). Optimal cut-offs determined from Youden’s index values showed that anti-CFP-10 antibody/IFN-γ ratios had a sensitivity of 48.5% (95% CI: 38.2–58.8) and a specificity of 89.7% (95% CI: 78.8–96.1), whereas anti-ESAT-6 antibody/IFN-γ ratios had a sensitivity of 54.6% (95% CI: 44.2–64.8) and a specificity of 75.9% (95% CI: 62.8–86.1) as shown in Table 3. This data shows that although the overall diagnostic potential as shown by area under ROC curve and specificity were improved when antibody/IFN-γ ratios were used to differentiate APTB from LTBI, the sensitivities of both biomarkers were significantly reduced.

**Table 3:** Potential of anti-CFP-10 and anti-ESAT-6 antibody/IFN-γ ratios to differentiate active and latent TB infection.

Biomarker	Cut-off	% Sensitivity (95% CI)	% Specificity (95% CI)	Youden's index (%)
**Anti-CFP-10 antibody/IFN-γ ratio**	512.0	48.5 (38.2–58.8)	89.7 (78.8–96.1)	38.1
**Anti-ESAT-6 antibody/IFN-γ ratio**	292.8	54.6 (44.2–64.8)	75.9 (62.8–86.1)	30.5

## 4.0 Discussion

This data showed that there is good correlation and agreement between antibody concentrations in QFT-GIT supernatants and sera from TB household contacts for both anti-CFP-10 & anti-ESAT-6 antibodies. The study also demonstrated good correlation and agreement between antibody concentrations in QFT-GIT nil and mitogen supernatants. It showed that anti-CFP-10 & anti-ESAT-6 antibodies in QFT-GIT supernatants when used independently detect TB disease in *M*.*tb* infected individuals with high sensitivity but have poor specificity and the reverse is true when these antibodies are used in combination with IFN-γ cytokine measurements.

The lack of any significant differences in concentrations of antibodies between sera and QFT-GIT nil supernatants, together with the strong correlation observed, suggests that serum anti-mycobacterial antibody concentrations can be approximated by QFT-GIT nil supernatant values in the TB household contacts studied. This finding was reassuring because we initially thought that there could be some differences as a result of accumulation of antibodies produced by *Mycobacterium* specific antibody secreting cells over the 16–24 h period of culture necessary for the QFT-GIT assays. However, we found no evidence of this effect. The reasons for agreement between serum and QFT-GIT supernatant antibody values may be because the QFT-GIT culture period is too short to allow accumulation of appreciable amounts of antibodies in the supernatants. It is important to mention however that the study participants used for these analyses were all healthy HHCs with no signs or symptoms of active TB. This result may therefore reflect the low levels of circulating antibody secreting cells in individuals without an active infection [24]. Studies measuring antibodies in lymphocyte supernatant assays suggest that there are higher levels of anti-mycobacterial antibodies following culture of cells from active TB cases [14]. However, we were not able to access matched serum and QFT-GIT supernatant pairs from active TB cases to investigate this effect in whole blood culture supernatants. This was because the parent study did not include collection of sera from APTB cases in its protocol, and is a limitation of our study. Our observation of agreement between QFT-GIT supernatant and serum antibody concentrations may therefore be limited to individuals without active disease, but is nevertheless an important finding.

There were strong and statistically significant correlations between anti-mycobacterial antibody concentrations in QFT-GIT nil and mitogen supernatants from our study group of uninfected individuals, those with LTBI and APTB cases. Further analysis showed no significant differences in antibody concentrations between these samples, indicating a good agreement. This implies that even highly potent cell stimulants such as PHA in QFT-GIT mitogen tubes may not have any significant effect on antibody levels over the 16–24 h period of culture. Furthermore, the lack of this effect seems to extend to the majority of the individuals studied, regardless of their TB infections status. This finding highlights the possibility of using QFT-GIT nil and mitogen supernatants interchangeably for antibody analyses.

Our study showed that concentrations of antibodies in QFT-GIT supernatants differed between individuals with APTB from LTBI. The observation of higher anti-CFP-10 and anti-ESAT-6 antibodies in APTB cases compared to individuals with LTBI is in keeping with prior findings from other studies [12,25,26]. Anti-CFP-10 antibodies differentiated APTB cases from individuals with LTBI with high sensitivity (~89%). This value was higher than that reported in a meta-analysis of the performance of purified *M*.*tb* antigens, including CFP-10, for the sero-diagnosis of TB [27]. Anti-ESAT-6 antibodies distinguished the two groups with a sensitivity of 71%. This was slightly higher than that reported in a study in The Gambia evaluating anti-ESAT-6 antibodies for sero-diagnosis [28]. However, the diagnostic specificity of both anti-CFP-10 and anti-ESAT-6 antibodies was low. The individuals with LTBI may have developed antibodies against the *M*.*tb* proteins assessed, resulting in a higher number of false positives and a decrease in the overall specificity. However, it is also important to note that individuals with LTBI are not a completely homogenous population. Results from positron emission tomography—computed tomography (PET-CT) scans indicate that this group may contain individuals at various stages of TB reactivation even in the absence of clinical symptoms of disease [29]. As expected, *M*.*tb* specific IFN-γ concentrations were higher in individuals with LTBI compared to those with APTB [30], a reverse of what was seen with the antibodies. We therefore explored antibody cytokine combinations in the form of *M*.*tb* specific antibody/IFN-γ ratios for use in the differentiation of APTB cases from individuals with LTBI. We showed that anti-CFP-10/IFN-γ ratio and anti-ESAT-6/IFN-γ ratios differed between the two groups and distinguished them with a high specificity (>75%). However, this approach resulted in a substantial decrease in sensitivity compared to when antibodies were used independently.

Our investigation of QFT-GIT supernatant antibodies and combinations of antibody & cytokine data as TB biomarkers was limited to only anti-CFP-10, anti-ESAT antibodies and IFN-γ. Study of more QFT-GIT supernatant antibody or antibody cytokine combinations may identify immunological markers capable of distinguishing APTB from LTBI with both high sensitivity and specificity. Such approaches have shown promise in the diagnosis of leprosy. A study by van Hooij *et al*. using lateral flow assays to detect cytokines and antibodies in whole blood culture supernatants showed that together these biomarkers helped distinguish lepromatous from tuberculoid forms of the disease [31]. Our data showing good correlation and agreement between antibody concentration in sera and QFN-GIT supernatants is an important finding because it means this approach can be used to assess both humoral and cellular responses, thereby reducing blood volumes.

In conclusion, our study shows that QFT-GIT supernatants can be used to substitute sera during the study of anti-TB antibody immunity in HHC of APTB cases. It shows that there is no effect of PHA stimulation in QFT-GIT whole blood cultures on antibody concentrations. Lastly, the study shows that anti-CFP-10 & anti-ESAT-6 antibodies in QFT-GIT supernatants differentiate APTB from LTBI with high sensitivity but have poor diagnostic specificity. The reverse is true when ratios of these antibodies and QFT-GIT IFN-γ cytokine measurements are used. More antibody or antibody/cytokine combinations need to be investigated to discover those with the best discriminatory power.

## Supporting information

S1 Fig Antibody concentrations in QFT-GIT nil control supernatants strongly correlate with those in QFT-GIT mitogen control supernatants regardless of TB infection. Panel A: anti-CFP-10 antibodies. Panel B: anti-ESAT-6 antibodies. The correlation coefficient (r) and the p values shown correspond to results from Spearman’s rank correlation.
(TIFF)

S2 Fig Bland Altman plots show no statistical differences in antibody concentrations between QFT-GIT nil and QFT-GIT mitogen control supernatants regardless of TB infection. The solid horizontal line represents the bias or average difference while the dotted horizontal lines are 95% confidence intervals. Panel A: anti-CFP-10 antibodies. Panel B: anti-ESAT-6 antibodies.
(TIFF)

S3 Fig Individuals with LTBI have higher concentrations of IFN-γ compared to APTB cases. Panel A: anti-CFP-10 antibody/IFN-γ ratio. Panel B: anti-ESAT-6 antibody/IFN-γ ratio. The Wilcoxon rank-sum test was used for comparisons.
(TIFF)

S1 Dataset QFT-GIT nil supernatant Vs. serum comparisons. (XLSX)

S2 Dataset QFT-GIT mitogen Vs. nil comparisons. (XLSX)
