# Supplementary figures and images for: Use of QuantiFERON®-TB Gold in-tube culture supernatants for measurement of antibody responses
Source: PLoS One. 2017 Nov 21;12(11):e0188396. doi: 10.1371/journal.pone.0188396 (PMC5697869; doi:10.1371/journal.pone.0188396)

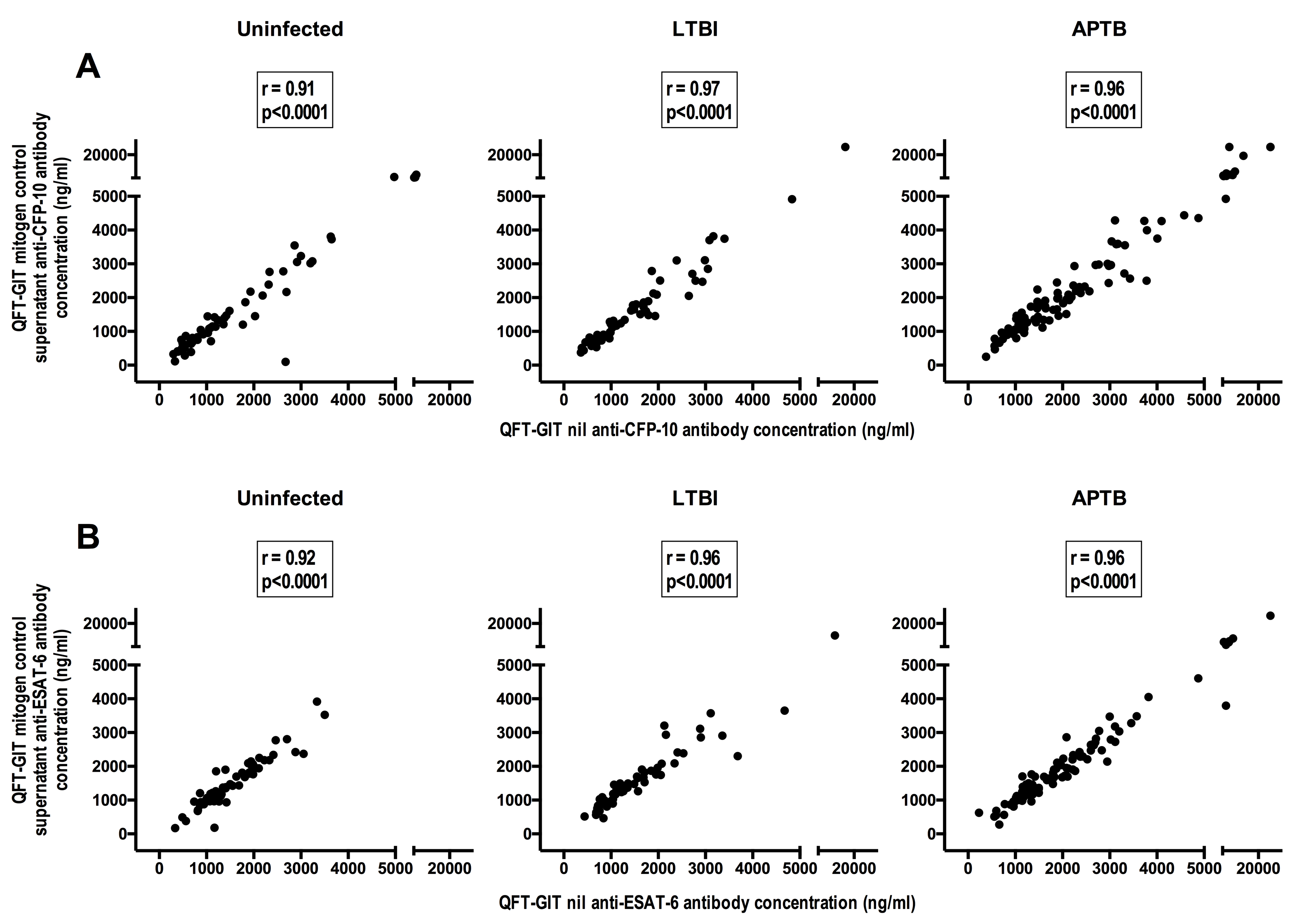

Supplement: S1 Fig — Panel A: anti-CFP-10 antibodies. Panel B: anti-ESAT-6 antibodies. The correlation coefficient (r) and the p values shown correspond to results from Spearman’s rank correlation. (TIFF) [file pone.0188396.s001.tiff]

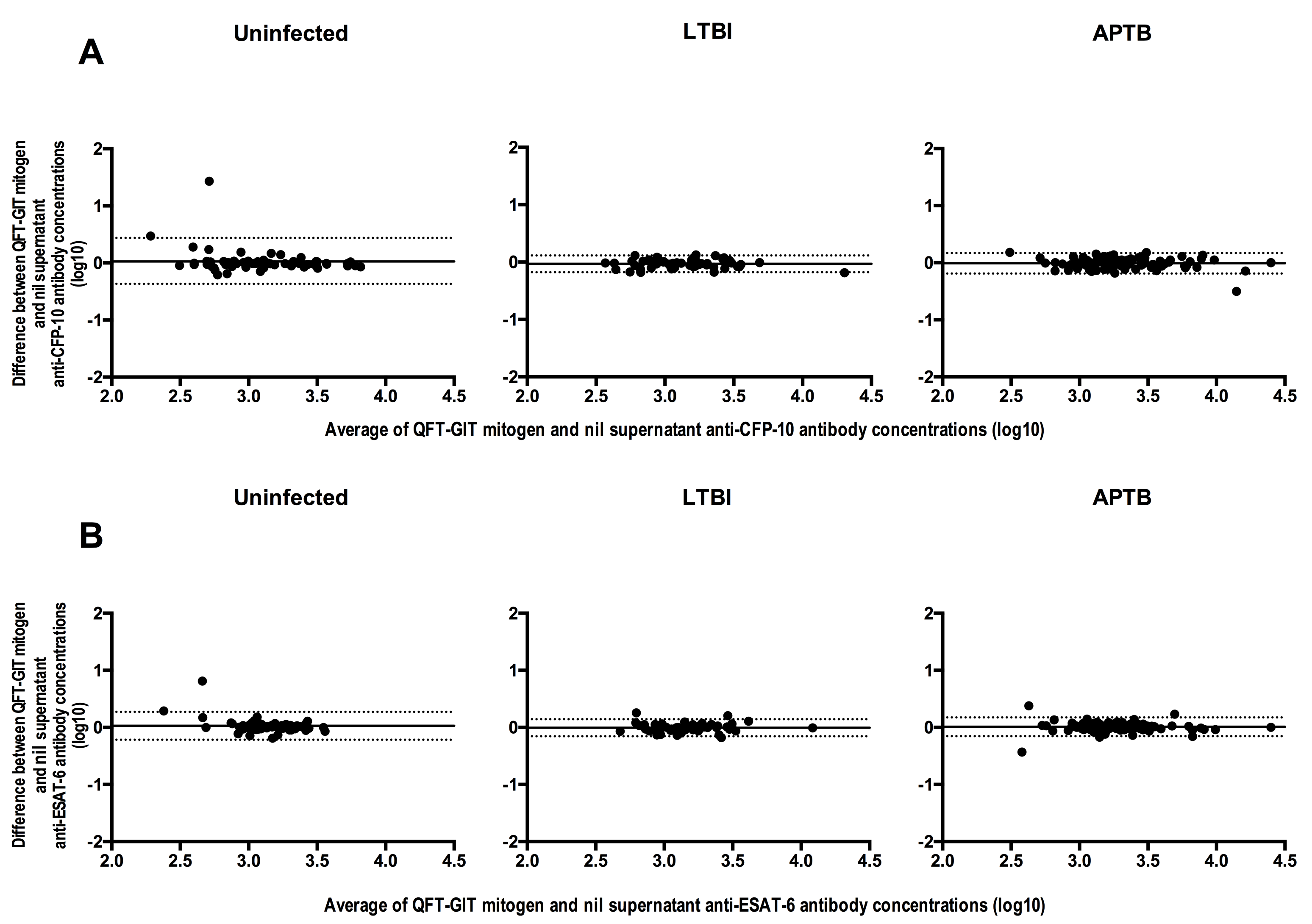

Supplement: S2 Fig — The solid horizontal line represents the bias or average difference while the dotted horizontal lines are 95% confidence intervals. Panel A: anti-CFP-10 antibodies. Panel B: anti-ESAT-6 antibodies. (TIFF) [file pone.0188396.s002.tiff]

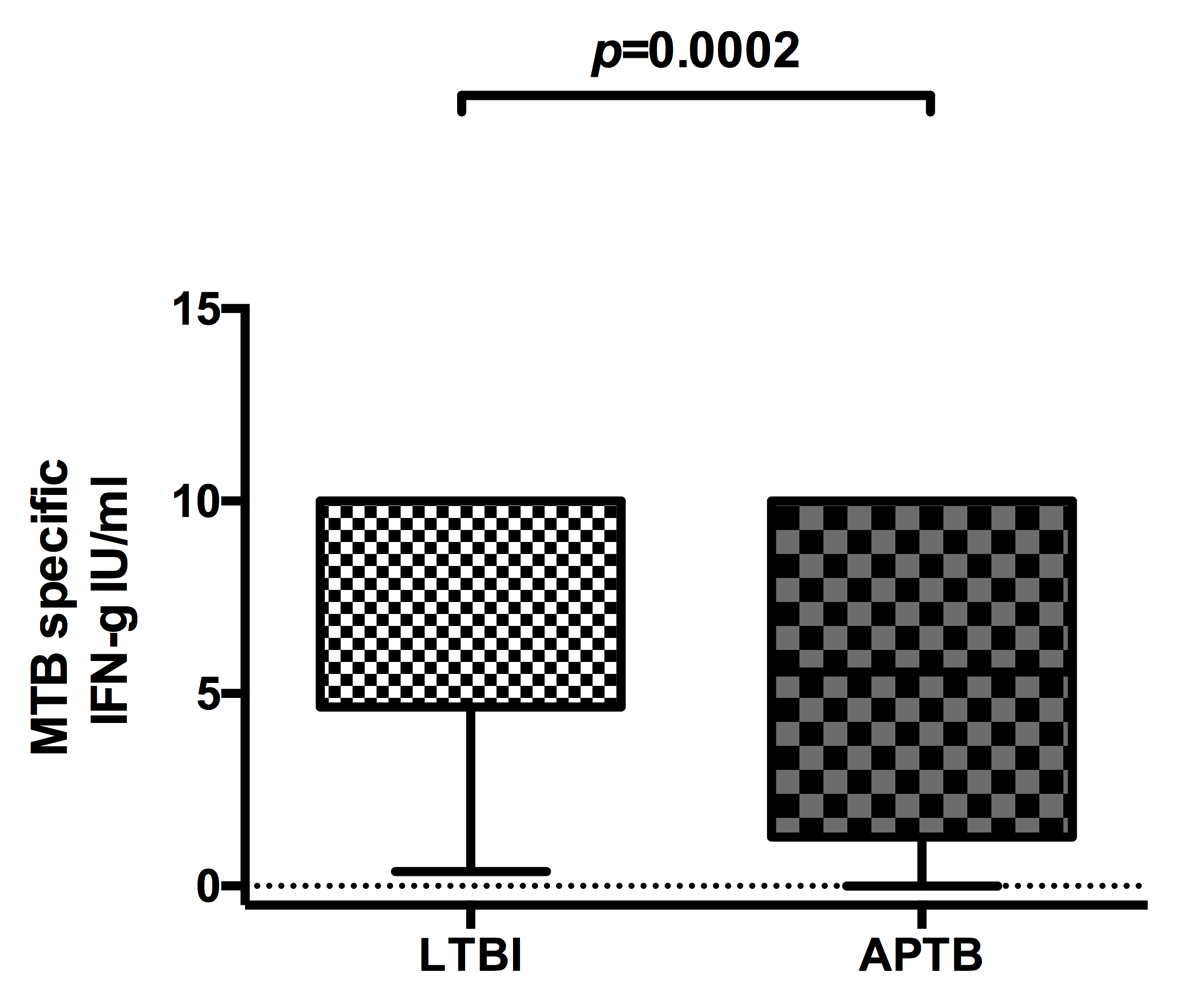

Supplement: S3 Fig — Panel A: anti-CFP-10 antibody/IFN-γ ratio. Panel B: anti-ESAT-6 antibody/IFN-γ ratio. The Wilcoxon rank-sum test was used for comparisons. (TIFF) [file pone.0188396.s003.tiff]
